# GSK3 acts as a switch for transcriptional programs in a model of low-grade gliomagenesis

**DOI:** 10.1186/s40478-025-02006-y

**Published:** 2025-04-30

**Authors:** Marilin S Koch, Minh Deo, Lena-Marie Schmitt, Michael S Hoetker, Şevin Turcan

**Affiliations:** 1 Clinical Cooperation Unit Neurooncology, German Consortium for Translational Cancer Research (DKTK), German Cancer Research Center (DKFZ), Heidelberg, Germany; 2 Department of Neurology and Neurooncology, University Hospital Heidelberg and National Center for Tumor Diseases, Im Neuenheimer Feld 400, 69120 Heidelberg, Germany; 3 Department of Internal Medicine V, Hematology, Oncology and Rheumatology, University Hospital Heidelberg, Heidelberg, Germany

**Keywords:** IDH mutation, Glioma, IDH^mut^-gliomagenesis, GSK3, WNT

## Abstract

Mutations in isocitrate dehydrogenase (IDH)1/2 are defining drivers of low-grade gliomagenesis. However, mutant IDH alone is not sufficient for malignant transformation, and additional events are required for the development of low-grade glioma. While specific genetic lesions have been identified to contribute to low-grade gliomagenesis, less is known about the signaling pathways involved in the acquisition of malignancy. To identify prerequisites of IDH mutant tumorigenesis, we modulated pathways previously implicated in glioma initiation using a tractable in vitro model system for early IDH1^R132H^-dependent gliomagenesis. Through the use of chemical compounds, we targeted WNT/GSK3, TGF-β and NOTCH-signaling, assessing their functional, transcriptional, and translational impacts. Expression of LGG-related marker L1CAM was affected by perturbation of all pathways, though only modulation of WNT/GSK3-signaling resulted in profound molecular transformation, including glioma-associated genes and programs regulating cellular architecture and cell replication. This was accompanied by altered cell morphology, migration capacity, and enhanced proliferation. Transcription factor RUNX2 was identified as a potential downstream effector, whose inhibition abrogated cell proliferation. Disrupted WNT/GSK3 signaling in a model system of early low-grade gliomagenesis fundamentally impacted cell fate, as demonstrated by a reshaped transcriptional landscape, aberrant transcription factor activity, extracellular matrix restructuring, and altered proliferation capacity. Our data suggests that GSK3 may play a central role during low-grade gliomagenesis, warranting further investigation.

**Supplementary Information:**

The online version contains supplementary material available at 10.1186/s40478-025-02006-y.

## Introduction

Gliomas are the most common primary malignant brain tumors [1] and are classified based on the presence or absence of isocitrate dehydrogenase (IDH) mutations into IDH wildtype (IDH^wt^) and IDH mutant (IDH^mut^) tumors, which are biologically distinct entities with significant differences in growth dynamics and prognosis.

Low-grade gliomas (LGGs) are defined as gliomas harboring an IDH mutation. Based on additional mutations, LGGs can be further distinguished into IDH^mut^ astrocytomas (mutations in *ATRX*,* TP53*, and *CDKN2A/B*) and oligodendrogliomas (codeletion of chromosomes 1p/19 and mutations in the *TERT* promoter, *CIC*,* FUBP1*, and *NOTCH1*) [2].

IDH1/2 mutations occur either at arginine 132 (IDH1) or 172 (IDH2) and induce broad epigenetic, transcriptional, and metabolic reprogramming. These mutations are considered initiating events in the development of LGGs [3]. However, the additional steps necessary to establish tumorigenic programs during low-grade gliomagenesis remain unclear, as does the role of specific signaling pathways involved in this process. This is largely due to limited model systems: establishment of syngeneic IDH^R132H^ glioma models has proven difficult. While IDH1^R132H^ knock-in was shown to successfully mimic gliomagenesis in vivo [4], implantation of patient-derived IDH1^R132H^ glioma tumorspheres is only feasible in immunocompromised animals, posing the added complication of a dysfunctional tumor microenvironment. Moreover, studying potential initiators of gliomagenesis is difficult with in *vivo* systems, as slow growth kinetics and limited transplantation efficiency hamper broad application.

In this study, we investigate the cell-intrinsic prerequisites of early IDH^mut^ gliomagenesis to improve our understanding of how these tumors develop and to identify pathways that could be targeted to abrogate malignant programs. To this end, we employ a tractable in vitro system to study key signaling pathways widely implicated in glial development and malignancy, using pharmacological inhibition.

Multiple developmental signaling pathways have been shown to significantly influence gliomagenesis [5–8]. However, previous studies largely focused on IDH^wt^ glioma. Since IDH^wt^ and IDH^mut^ gliomas are biologically distinct entities, differing in their genetic make-up, tumor composition, dynamics, and prognosis, findings in IDH^wt^ gliomas cannot be directly translated to IDH^mut^ gliomagenesis. Indirect evidence suggests that NOTCH, TGF-β, and GSK3 signaling may play roles in this context, as pathways members or downstream effectors have been demonstrated to be differentially expressed upon IDH mutation in the model system used in this study [9]. Furthermore, an integrative transcriptional analysis of TCGA and REMBRANDT datasets revealed increased NOTCH signaling in the IDH^mut^ non-codel cohort [10]. NOTCH signaling has been shown to play an instructive role in gliomagenesis [11].

TGF-β signaling plays an important role in gliomagenesis by enhancing the proliferative, invasive, angiogenic, and immunosuppressive capacities of glioma cells, while also promoting stemness features [3]. While most research has focused on its role in glioblastoma, recent studies show that LGGs can be classified into subtypes based on immune cell infiltration and prognosis, determined by the expression of TGF-β related genes [12].

WNT signaling, another key developmental pathway, has been implicated in the development of multiple tumor entities [13], including low-grade gliomas [14, 15]. The serine/threonine kinase GSK3 is a crucial component of canonical WNT signaling and is also implicated in other pathways that potentially impact LGG development, such as EGFR, RAS, PI3K, PTEN, AKT, mTOR and NFκB [16]. As a master regulator of cytoskeletal architecture, GSK3 signaling is essential for development and differentiation [17]. Its dysregulation is associated with a range of disorders, from neuropsychiatric conditions to metabolic diseases and cancers [18], including gliomas [19, 20]. GSK3 interaction partners PI3K/AKT/mTOR are specifically connected to IDH^mut^ tumorigenesis [21, 22], while expression of WNT pathway members has been shown to decrease upon IDH1^R132H^ [23].

In this study, we aim to determine the cell-intrinsic effects of WNT/GSK3, TGF-β, and Notch signaling in a tractable model of early IDH1^R132H^ gliomagenesis using chemical compound-mediated disruption. Notably, modulation of WNT/GSK3 signaling induced profound differential expression of LGG-associated genes and programs, resulting in altered migratory capacity and increased cell proliferation. Through pharmacological targeting, we identified the transcription factor RUNX2 as a potential effector of this phenotype. RUNX2 expression is associated with reduced survival in LGG patients, highlighting its relevance as a potential therapeutic target warranting further investigation.

## Materials and methods

### Cell culture

Inducible IDH1^R132H^ expressing immortalized human astrocytes were kindly provided from Timothy A. Chan (Cleveland Clinic). Generation of this doxycycline-inducible model system has been described previously [9]. Cells were maintained in high glucose DMEM (Gibco) with 10% FBS and 1% penicillin/streptomycin. 1 µg/ml Doxycycline was added to the medium every 72–96 h to ensure expression of IDH1^R132H^. For parallel inhibition of signaling pathways, 3 µM CHIR99021 (targeting GSK3; Axon Medchem), 5 µM Repsox (targeting TGFβ-ALK5; Sigma-Aldrich) or 10 µM YO-01027 (targeting γ-secretase; Selleckchem) were added to the medium in accordance with previous publications focusing on pathway modulation [24–26]. DMSO served as vehicle control. Experimental procedures were started after 40 days of treatment.

### RNA-Seq

For RNA-Seq, 2 days after concomitant treatment with doxycycline and compounds targeting GSK3, TGFβR-1/ALK5 or γ-secretase, RNA was isolated in triplicates with the Rneasy Mini Kit (Qiagen). For RNA-Seq following siRUNX2 treatment in GSK3-treated cells, RNA was isolated after 3 days of incubation. Library preparation and sequencing with NovaSeq 6000 in 50 or 100 bp paired-end modewas performed at the NGS Core Facility at DKFZ Heidelberg. Analysis for differentially expressed genes was performed in R utilizing featureCounts and edgeR. AI was used for code debugging.

### Mass spectrometry

Inducible IDH1^R132H^ expressing immortalized human astrocytes were seeded in technical quadruplicates and treated for 72 h as indicated, followed by protein isolation. Samples were submitted for mass spectrometry utilizing an Orbitrap Exploris 480 mass spectrometer at the DKFZ GPCF-MS-based Protein Analysis Unit. Analysis of raw wasperformed with MaxQuant (version 2.1.4.0); subsequent quantification and statistical analysis were carried out using the MaxLFQ algorithm [27] and the limma package in R respectively. The Benjamini-Hochberg method was applied for multiple testing correction.

### Immunofluorescence

10,000 cells/well were seeded in triplicates on coverslips in a 24-well plate and incubated with doxycycline and CHIR99021, Y-27,632, or vehicle control for 24 h. Cells were fixed with 4% paraformaldehyde (PFA), permeabilized with 0.1% Triton X-100, and incubated overnight at 2–8 °C with the following primary antibodies: Phalloidin-iFluor 594 (abcam, 176757, 1:1000) and Paxillin (Rabbit mAb, abcam, 32084, 1:50). This was followed by one hour incubation with the secondary antibody (Alexa Fluor 488 donkey anti-rabbit, ThermoFisher 1:2000) and a 5 min incubation with DAPI (1 µg/ml) at room temperature. Coverslips were mounted using VectaShield Vibrance Antifade Mounting Medium (Biozol). Images were acquired with Olympus VS200 Slideview Scanner at DKFZ and subsequently processed using QuPath [28].

### Live cell microscopy

IDH^mut^ immortalized human astrocytes were seeded in 6-well plates and treated with CHIR99021. After 24 h, images of cells were acquired at 10× magnification using a NIKON Ti Eclipse microscope. Cell diameter was measured utiziling Fiji/ImageJ [29].

### Flow cytometry

Cells were treated for 72 h in 6-well plates in triplicates. After trypsinization and subsequent washing, staining for L1CAM/AF647 (Biolegend, 3716068,) was performed at room temperature. Zombie live/dead violet dye (Biolegend, 423113) was used to discriminate dead cells. Cells were then fixed with 4% PFA. Flow cytometry was carried out utilizing a BD Canto within the DKFZ FACS core facility, followed by data analysis with FlowJo (BD Bioscoences).

### EdU cell cycle analysis

Cell cycle analysis after treatment with CHIR99021, CHIR99021/siRUNX2, and the respective controls was performed using the Click-iT™ Plus EdU Alexa Fluor™ 647 Flow Cytometry Assay Kit (Thermo Fisher, C10634) according to the manufacturer’s recommendations. Nuclear counterstaining was carried out with DAPI. Flow cytometry was performed with a BD Fortessa at the DKFZ FACS core facility and resulting data analyzed with FlowJo (BD Biosciences).

### Clonogenicity assays

Immortalized IDH^mut^ human astrocytes were seeded at 1000 cells/ well in 6- or 12-well plates and treated immediately. After incubation for 7–10 days at 37 °C, plates were washed with PBS, fixed with 4% PFA and stained with 0.5% crystal violet. Images were taken with the Epson Perfection V850 Pro Scanner at the DKFZ Light Microscopy Facility. Further analyses were carried out in Fiji with either the Cell Counter plugin or the Colony Area plugin [30].

### Migration assay

Migration assays of cells modulated with CHIR99021 or vehicle control were performed with the Cell Migration/Chemotaxis Assay Kit (abcam, ab235673) according to the manufacturer’s recommendations.

### Scratch assay

On day 0, 15,000 cells were seeded in a 96-well plate in quadruplicates and treated with either CHIR99021 or control and doxycycline in complete DMEM as indicated. On day 1, a scratch wound was created using a wound maker (Essen Bioscience).The cells were then washed twice with PBS and maintained in DMEM containing above mentioned compounds for the remainder of the experiment. Automated image acquisition was performed with the IncuCyte S3 (Sartorius) every 2 h for 48 h. Two images per well were captured at different positions. Scratch wound width was analyzed with the “MRI Wound Healing tool” in Fiji. Statistical testing was performed with non-linear regression analysis in GraphPad Prism.

### qPCR

RNA was isolated with the Rneasy Mini Kit (Qiagen), followed by cDNA synthesis with either iScript cDNA synthesis kit (BioRad) or high-capacity RNA-to-cDNA-kit (applied biosystems). RNA and cDNA concentrations were measured with a Nanodrop. qPCR was performed with Power SYBR Green PCR Master Mix (applied biosystems) on a LightCycler 480 with primers for *RUNX2* (KiCqStart™, Merck) and *GAPDH* (F: AAGGTGAAGGTCGGAGTCAA, R: AATGAAGGGGTCATTGATGG).

### RNA interference

For RNA interference experiments siRNA targeting RUNX2 (Horizon Discovery, ON-TARGETplus Human SMARTPool, #L-012665-00-0005) and a Non-Targeting Pool siRNA (Horizon Discovery, ON-TARGETplus Human SMARTPool, #D-001810-10-05) as control were used. siRNAs were resuspended in 1x siRNA Buffer (Horizon Discovery, B-002000-UB-100). For all applications, cells were treated with 23 nM siRNA. For isolation of RNA from CHIR99021-treated cells after siRNA-mediated RUNX2-knockdown, 10,000 cells were seeded in a well of a 12-well plate, treated with 23nM and harvested after 72 h.

### Statistics

GraphPad Prism (version 10) was used to determine statistical significance with Student’s *t*-test, unless stated otherwise.

## Results

### Inhibiting GSK3 with CHIR99021 significantly alters expression of the LGG marker L1CAM in a model of early low-grade gliomagenesis

**Fig. 1:**
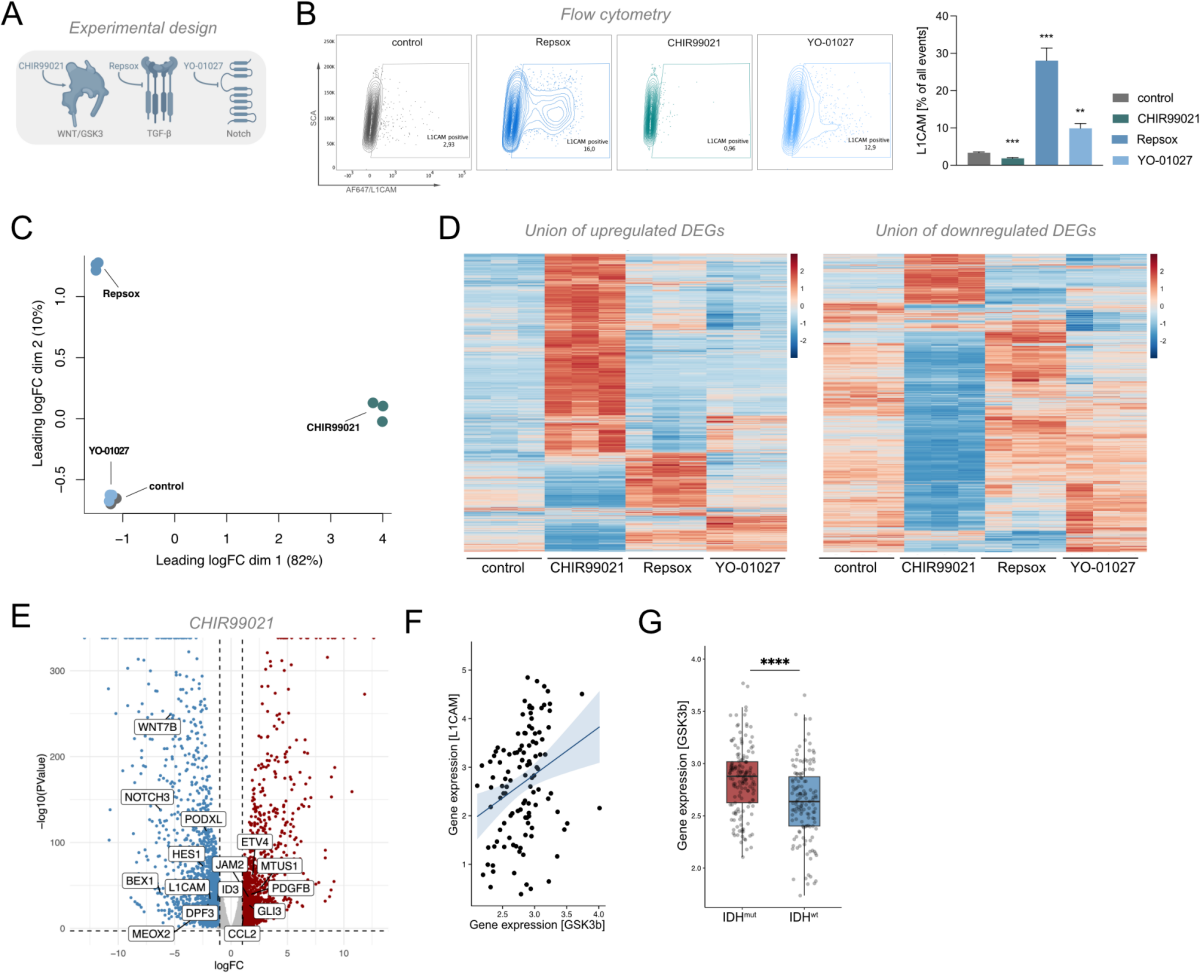
GSK3 inhibition disturbs IDH1^R132H^ induced transcriptional programs. **A** Experimental Design. Chemical inhibition of WNT/GSK3 (CHIR99021), TGF-β (Repsox) and γ-secretase (YO-01027) in immortalized conditional IDH1^R132H^ astrocytes. **B** Flow cytometry after treatment with CHIR99021, Repsox and YO-01027 showed significantly changed expression of LGG-associated marker L1CAM (*n* = 2 biological and 3 technical replicates each). **C** Multidimensional scaling analysis of RNA-seq data showing segregation of CHIR99021- and Repsox-treated samples from control samples. **D** Heatmaps of all differentially expressed genes upon treatment with CHIR99021, Repsox, or YO-01027, showing most profound transcriptional reshaping after GSK3 inhibition. **E** Distribution of up- and downregulated DEGs after GSK3 inhibition, showing a higher fraction of downregulated genes, including those relevantin IDH^mut^ gliomas. **F** Gene expression of *L1CAM* and *GSK3B* is positively correlated in IDH^mut^ glioma patient cohorts (*R* = 0.29, *p* = 0.0017) (source: CGGA mRNA dataset, only primary IDH^mut^ glioma from cases included). Correlation was determined with Pearson’s correlation test. **G*** GSK3B* is significantly overexpressed in IDH^mut^ gliomas compared to IDH^wt^ gliomas (Wilcoxon test, *p* = 5.9 × 10^− 7^; source: CGGA)

To assess the contribution of glioma-relevant pathways to IDH-mutant gliomagenesis, immortalized human astrocytes conditionally expressing IDH1^R132H^ were treated simultaneously with doxycycline for IDH1^R132H^ induction and the following compounds: CHIR99021 targeting GSK3/WNT signaling, YO-01027 targeting γ-secretase/NOTCH signaling, and Repsox targeting ALK5/TGF-β signaling to modulate the respective pathways (Fig. 1a). Expression levels of the cell adhesion marker L1CAM were measured with flow cytometry to determine early effects of pharmacological treatment on low-grade gliomagenesis, as L1CAM has been shown to be upregulated in IDH1^R132H^ astrocytes [9], and in IDH1^R132H^ glioma patients (source: CGGA). While inhibition of GSK3 led to significantly decreased L1CAM expression (*p* = 0.001–0.024), the opposite was observed with ALK5 and γ-secretase inhibition (Repsox: *p* = 0.0001–0.0002, YO-01027: *p* = 0.0002–0.0011) (Fig. 1b). TGF-β1 was previously shown to reduce L1CAM expression in a model of pancreatic ductal adenocarcinoma, paralleling our findings [31]. γ-Secretase has so far been implicated in the further processing of ADAM10-cleaved L1CAM [32].

### GSK3 Inhibition profoundly alters transcriptional programs in IDH1^R132H^ astrocytes

To gain insight into the consequences of disrupted signaling cascades controlled by GSK3, ALK5 and γ-secretase on the IDH1^R132H^ shaped transcriptome, we performed RNA-seq. Multidimensional scaling analysis showed a distinct separation of samples treated with Repsox and CHIR99021 from controls, which was not the case for y-secretase treated samples (Fig. 1c). To better understand the differential transcriptional impact of these compounds, we conducted a comparative analysis of all differentially expressed genes (DEGs) and observed profound transcriptional changes upon GSK3 inhibition with CHIR99021 (Fig. 1d). Our analysis identified 1487 significantly upregulated and 1929 significantly downregulated genes (FDR < 0.05, abs(logFC) > 1), including alterations in numerous LGG-associated genes (Fig. 1e). Genes that are upregulated in IDH^mut^ glioma, but are downregulated after CHIR99021 treatment included *WNT7b*,* L1CAM*,* BEX1*, and *ID3*, while genes that show downregulation in IDH^mut^ glioma, but are upregulated after CHIR99021 included *PDGFB*,* GLI3, and CCL2.* TGF-β and γ-secretase inhibition showed a less substantial impact on the transcriptional landscape (Fig. 1d, Suppl. Fig. S1a, b).

These results suggest that GSK3 plays a crucial role in establishing LGG-relevant transcriptional programs. Indeed, our interrogation of publicly available patient expression data from LGG patients confirmed a positive correlation between *L1CAM* and the predominant isoform *GSK3B* in IDH^mut^ gliomas (Fig. 1f, source: CGGA) and showed that *GSK3B* is significantly upregulated in IDH^mut^ glioma patients (Fig. 1g, source: CGGA).

### Modulation of IDH1^R132H^ astrocytes with CHIR99021 results in morphological transformation and reduced migratory capacity

**Fig. 2:**
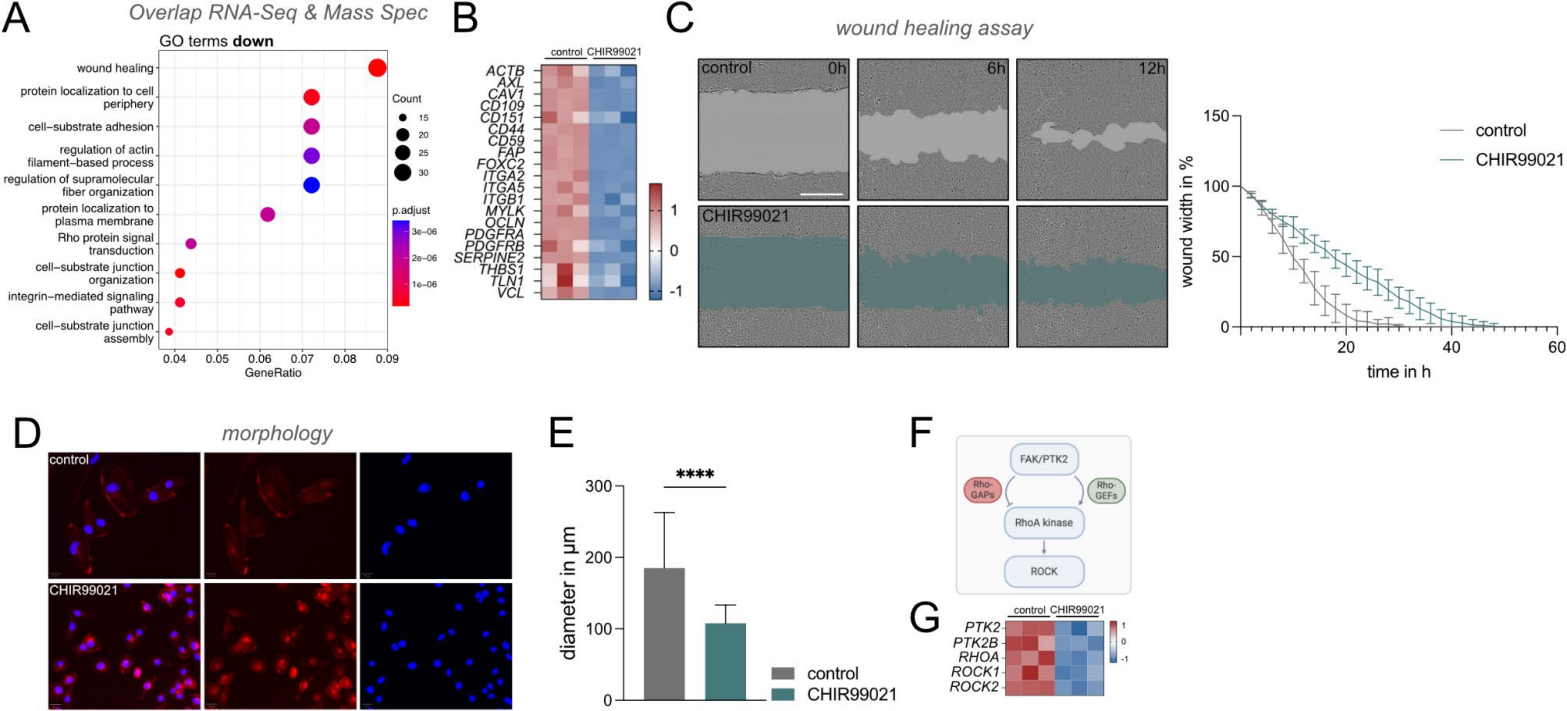
Reduced migration and altered morphology upon GSK3 inhibition. **A** Gene ontology analysis of intersected RNA-seq and mass spectrometry data demonstrates significant enrichment for wound healing, cell adhesion and ECM related terms among downregulated genes and proteins after GSK3-inhibition with CHIR99021. **B** Heatmap of selected candidates within the GO wound healing family. *PDGFRA* is among the significantly downregulated genes affecting migratory capacity. **C** After inflicting a scratch wound, CHIR99021-treated IDH1^R132H^ astrocytes display decreased migration capacity over time, as shown in representative images. Wound width at different timepoints highlighted in grey (control) and light green (CHIR99021. Scale bar = 400 μm. Statistical analysis confirms that wound healing (migration) was significantly reduced after CHIR99021 treatment (non-linear regression curve analysis, *p* < 0.0001–0.0356) (*n* = 3 biological and 4 technical replicates each). **D** Immunofluorescence with DAPI (blue) and Phalloidin (red) staining demonstrates altered cell morphology upon GSK3 inhibition with reduced cell size and cell spreading. Scale bar = 20 μm. (*n* = 3 technical replicates). **E** Comparison of maximum cell diameters shows a significantly reduced cell diameter after treatment with CHIR99021 (*p* < 0.0001) (*n* = 3 biological replicates). **F**,** G** Simplified schematic of FAK/RhoA/ROCK interaction and corresponding heatmap of the respective genes upon modulation with CHIR99021

To assess the consequences of the transcriptional alterations in IDH1^R132H^ astrocytes, we performed mass spectrometry and intersected these data with our RNA-seq datasets, initially focusing on downregulated genes and proteins. Here, gene ontology analysis showed significant enrichment of the GO term “wound healing” as a top candidate and multiple GO families associated with ECM restructuring, including Rho signaling (Fig. 2a, b). Consistent with these findings, treatment with CHIR99021 led to a significantly reduced wound healing capacity in IDH1^R132H^ astrocytes (*p* = 0.0356 – *p* < 0.0001) (Fig. 2c). These results were confirmed with a Boyden chamber-based migration assay (Suppl. Fig S2a), demonstrating consistent reduction in migratory potential.

Additionally, cell morphology of IDH1^R132H^ astrocytes was strikingly altered upon GSK3 modulation, resulting in smaller, rounded cells (Fig. 2d) with a significantly reduced maximum cell diameter compared to controls (*p* < 0.0001) (Fig. 2e), consistent with GO terms associated with cytoskeleton and ECM attachment. Correspondingly, we observed downregulation of Vinculin (Fig. 2b), whose knockdown has been shown to diminish cell spreading, resulting in rounded cells [33, 34]. Of note, reduced GSK3 expression has been demonstrated to impair migration in HeLa cells [35], and GSK3 is known to regulate cytoskeletal organization and cell migration through interactions with focal adhesion proteins (FAK), which in turn modulate ROCK activity through RhoA kinase [36, 37] (Fig. 2f) [37, 38]. Indeed, our transcriptional data show downregulation of FAK/RhoA/ROCK signaling targets, pointing to this pathway as a potential downstream effector of the observed phenotype (Fig. 2g). We also identified significant downregulation of *PDGFRA* upon CHIR99021 treatment in our model, a gene implicated in wound healing (Fig. 2b). PDGFRA is a receptor tyrosine kinase that integrates growth factor stimuli and whose overexpression in LGGs is directly induced by the IDH mutation [39]. Our data demonstrates significant downregulation of *PDGFRA* at both the transcriptional and protein levels in our RNA-seq and mass spectrometry datasets after GSK3 inhibition (Suppl Fig S2b, c), suggesting a potential regulatory relationship between *GSK3* and *PDGFRA*. Collectively, our data shows that GSK3 inhibition interferes with key migratory phenotypes associated with IDH^mut^ in LGGs. This aligns with the initially observed reduction in L1CAM expression, as pathways mediating ECM contact are strongly impacted by transcriptional downregulation.

### Increased proliferation upon GSK3 Inhibition in IDH1^R132H^ astrocytes

**Fig. 3:**
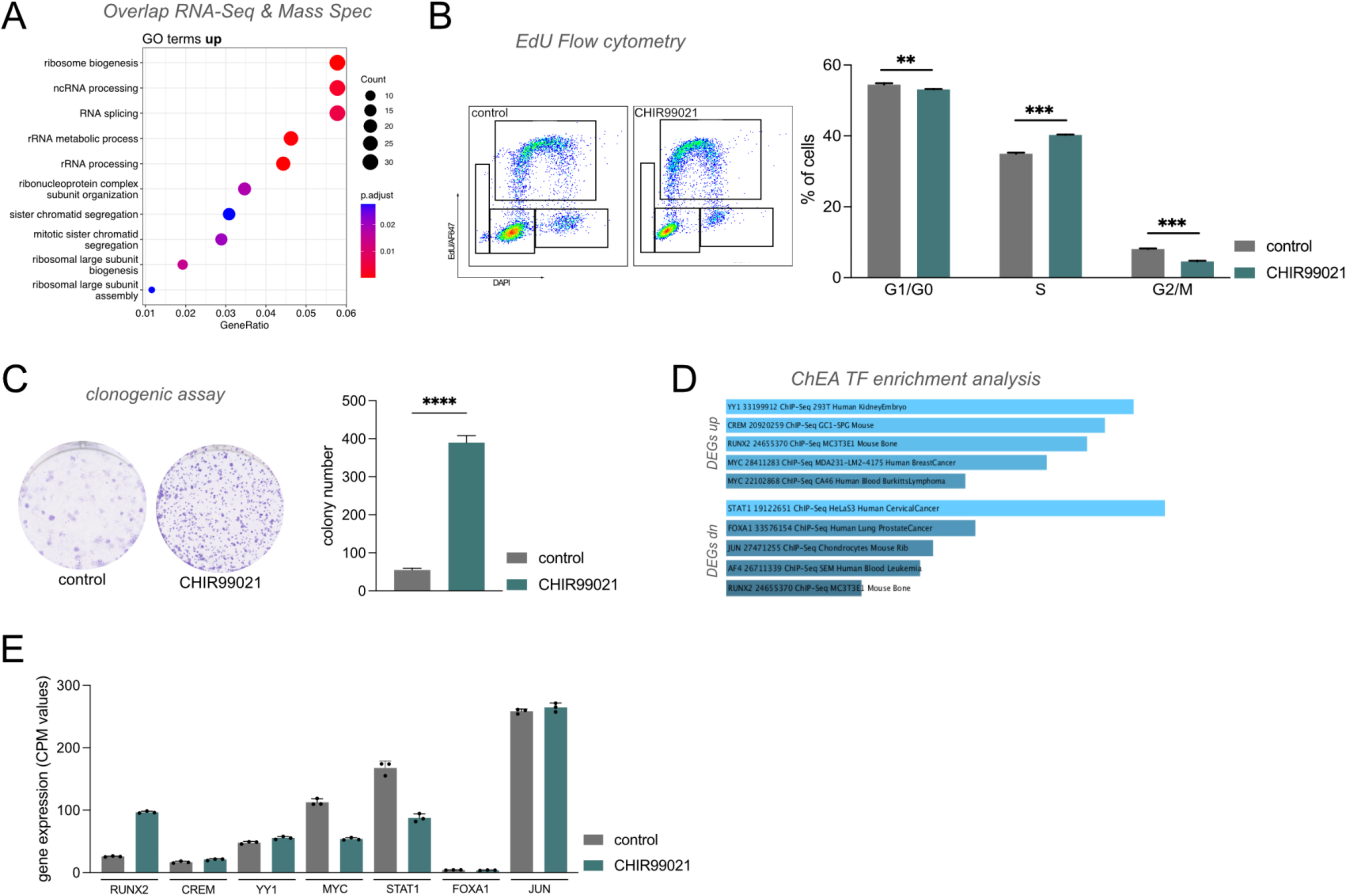
Increased proliferative capacity and clonogenicity after GSK3 inhibition is associated with RUNX2 overexpression. **A** Gene ontology analysis of intersected RNA-seq and mass spectrometry data demonstrates significant enrichment for terms associated with cell proliferation among upregulated genes and proteins upon CHIR99021 treatment. **B** Cell cycle analysis using EdU flow cytometry showing a significant increase in S-phase (*p* = 0.0008–0.00001) along with a significant reduction of cells in G0/G1 (*p* = 0.00153–0.002919) and inconsistent reduction of G2/M (p = ns – 0.000025), indicating increased cell cycling (*n* = 3 biological and 3 technical replicates each). **C** Treatment of IDH1^R132H^ astrocytes with CHIR99021 significantly enhances clonogenic proliferation (*p* < 0.0001 – *p* = 0.0024) (*n* = 3 biological and 3 technical replicates each). **D** EnrichR transcription factor analysis reveals RUNX2 among the top 5 transcription factors regulating the expression of both up- and downregulated DEGs. **E** CPM expression profiles of the top transcription factors identified with EnrichR emphasizes RUNX2 as the only transcription factor significantly upregulated upon GSK3 inhibition

Focusing on upregulated genes and proteins, we detected an enrichment of GO terms associated with augmented transcriptional activity (Fig. 3a), indicating an enhanced proliferative state with accelerated protein biosynthesis. Correspondingly, cell cycle analysis showed a significantly increased fraction of cells in S phase (*p* = 0.0008–0.00001) and a significantly reduced number of cells in G0/G1 phase (*p* = 0.00153–0.002919). These findings were paralleled by a significant increase in clonogenicity (*p* < 0.0001 – *p* = 0.0024) (Fig. 3c). Although certain stem cell markers (e.g. CD44, ALDH1L1) were downregulated, the observed increase in clonal proliferation is likely not linked increased stem cell population. A potential mechanism explaining the increased clonal proliferation capacity could involve WNT activation secondary to GSK3 inhibition, which has previously been associated with increased clonal proliferation.

In contrast to the initially observed reduction in the LGG marker L1CAM, these results discussed above a more complex impact of GSK3 inhibition in maintaining pro-tumorigenic transcriptional programs. To better understand how this shift from a migratory, ECM-interacting cell state to a highly proliferative and clonogenic phenotype is mediated, we aimed to identify upstream drivers potentially responsible for this transcriptional rewiring. To this end, we performed transcription factor enrichment analysis utilizing ChIP-X enrichment analysis (ChEA) with EnrichR [40–42], separately analyzing up- and downregulated DEGs. With this approach, we identified seven transcription factors (*YY1*, *CREM*, *RUNX2*, *MZC*, *STAT1*, *FOXA1*, *JUN*) potentially regulating the observed transcriptional changes. Notably, the transcription factor *RUNX2* was among the top regulators of both up- and downregulated genes (Fig. 3d) and was the only transcription factor significantly upregulated upon GSK3 inhibition in our dataset (Fig. 3e).

### RUNX2 is a potential regulator of cell fate change upon GSK3 Inhibition in IDH1^R132H^ astrocytes

**Fig. 4:**
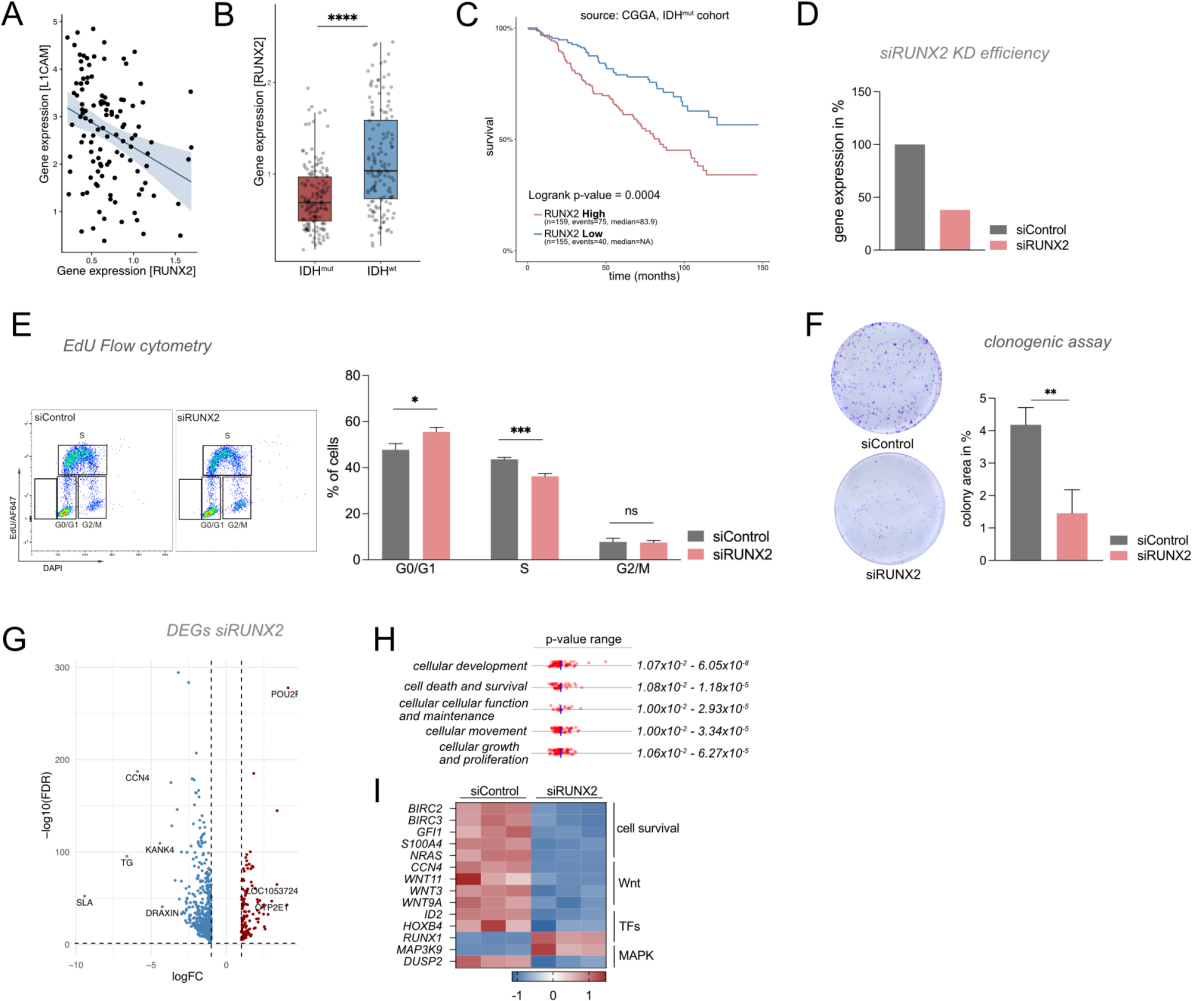
RUNX2 knockdown in CHIR99021 treated cells decreases proliferation and clonal expansion potential. **A** Negative correlation of *RUNX2* and *L1CAM* expression in IDH^mut^ glioma patients (*R* = –0.313, *p* = 0.000648) (source: CGGA, mRNA dataset; only primary IDH^mut^ gliomas included). Correlation was determined using Pearson’s correlation test. **B*** RUNX2* is significantly overexpressed in IDH^wt^ compared to IDH^mut^ gliomas (*p* = 8.5 × 10^− 11^) (source: CGGA). **C** High *RUNX2* expression is associated with significantly impaired survival in patients with IDH^mut^ gliomas (source: Gliovis/CGGA). **D** qPCR analysis of *RUNX2* expression after siRNA-mediated knockdown in CHIR99021-treated IDH^mut^ astrocytes shows reduced *RUNX2* expression compared to control (*n* = 2 biological and *n* = 3 technical replicates per sample). **E** Cell cycle analysis with EdU flow cytometry after siRNA-mediated knockdown of RUNX2 in CHIR99021-treated cells demonstrates a significant decrease of cells in S phase (*p* = 0.0002–0.0047), a significant increased proportion of cells in G0/G1 (*p* = 0.0013–0.0161), and inconsistently enhanced proportions of cells in G2/M (*p* = 0.0331 – ns), (*n* = 3 biological and 3 technical replicates each). **F*** RUNX2* knockdown in CHIR99021-treated astrocytes leads to significantly reduced clonal expansion potential (*p* = 0.0062–0.0182) (*n* = 3 biological and 3 technical replicates each). **G** Distribution of up- and downregulated DEGs after *RUNX2* knockdown in CHIR99021-treated IDH^mut^ astrocytes, showing a higher fraction of downregulated genes, including several relevant in IDH^mut^ gliomas. **H** Ingenuity Pathway analysis of *RUNX2*-dependent DEGs highlightssignificant enrichment for proliferation-relevant functions, including cellular development, cell death and survival, and cellular growth and proliferation. **I** Heatmap of selected genes associated with cell death and survival from the siRUNX2 RNA-seq dataset

RUNX2 is a transcription factor involved in skeletal development [43], and while its overexpression is associated with elevated metastatic potential in breast cancer [44], its implications for low-grade gliomagenesis are unclear. To better understand its potential clinical relevance, we analyzed publicly available expression data of glioma patient samples with matched clinical data. In line with our observations, *RUNX2* expression negatively correlates with *L1CAM* expression (Fig. 4a). Furthermore, RUNX2 is typically expressed at higher levels in IDH^wt^ compared to IDH^mut^ gliomas (Fig. 4b). However, within the IDH^mut^ glioma patients, high *RUNX2* expression is associated with severely impaired overall survival (Fig. 4c), a pattern not observed in IDH^wt^ glioma patients (Suppl. Fig S3). To dissect how RUNX2 might mediate these LGG-specific findings and contribute to the phenotypes observed in our model system, we used RNA interference to knock down *RUNX2* in conjunction with CHIR99021 treatment in IDH1^R132H^ astrocytes (Fig. 4d). Indeed, cell cycle analysis revealed a significantly reduced proportion of cells in S phase (*p* = 0.0002–0.0047) together with an increased fraction of cells in G0/G1 phase (*p* = 0.0013–0.0161), suggesting decreased cell cycling upon *RUNX2* knockdown (Fig. 4e). Furthermore, the increased clonogenicity of CHIR99021-treated cells was significantly reduced upon RUNX2 knockdown (*p* = 0.0062–0.0182) (Fig. 4f). To pinpoint downstream targets of *RUNX2* potentially mediating the observed cell fate change, we performed RNA-seq after inhibition of GSK3 alone and in combination with *RUNX2* knockdown. After filtering for significance (|logFC| > 1, FDR < 0.05), we detected a total of 664 genes, with 544 genes showing decreased expression and 120 showing increased expression levels after RUNX2 knockdown (Fig. 4g). Functional enrichment analysis of these DEGs revealed significant overrepresentation of proliferation-associated terms (Fig. 4h). A detailed assessment of genes clustering within these functional families indicates that the RUNX2-associated phenotype is not mediated unilaterally but rather the product of a complex interplay of diverse signaling cascades including, but not limited to, WNT and MAP kinase pathways, both of which are implicated in gliomagenesis [16] and canonical regulators of GSK3.

## Discussion

In this study, we demonstrate that GSK3 inhibition fundamentally affects cellular programs in a model system of IDH^mut^ gliomagenesis by profoundly reshaping the transcriptional landscape. While ECM remodeling and migratory capacities were negatively affected, we observed higher clonogenicity and proliferation. Our experiments identify RUNX2 as a transcription factor downstream of GSK3 activity that mediates a pro-proliferative cell state and whose increased expression is associated with impaired survival in IDH^mut^ glioma patients.

Leveraging a tractable in vitro system of IDH^mut^ low-grade gliomagenesis, we identified GSK3 signaling as a crucial regulator for IDH^mut^ specific transcriptional programs. GSK3 notably regulates the expression of transcription factos, which orchestrate large transcriptional programs [18]. This is highlighted by the broad transcriptional modulation observed upon GSK3 inhibition in our model system. *GSK3 i*s highly expressed in neuronal tissue [16] and plays a critical role during neurogenesis [45]. Its deregulation directly contributes to neurodegenerative, psychiatric, and neoplastic disease [18] and has been shown to affect proliferation in glioblastoma models [20]. Together with the fact that the predominant isoform *GSK3B* is upregulated in IDH^mut^ glioma (CGGA) and astrocytes [46], these findings in combination with our data support the notion that GSK3 signaling plays an important role during IDH^mut^ gliomagenesis. As the model used in this study has been extensively characterized and successfully applied to assess various aspects of low-grade gliomagenesis, it provides a robust foundation for further studies, including in vivo experiments that would also enable the exploration of cell-extrinsic effects and the potential impact on the tumor microenvironment.

Inhibition of GSK3 led to reduced expression of cell adhesion molecule L1CAM, which is overexpressed both in IDH^mut^ glioma and in our model system (CGGA [9]). This was accompanied by decreased expression of genes and proteins associated with ECM remodeling and migration, resulting in reduced migratory capacity. ECM and cytoskeletal organization are fate-determining factors for gliomas by affecting morphology, migration, invasion, and proliferation [47], and are particularly relevant in the context of IDH mutant biology [48]. The reduced migratory potential observed following GSK3 inhibition in our study aligns with previous studies on genetic and chemical inhibition of GSK3 in other cell types [49]. Furthermore, our data indicate a connection between GSK3 and PDGFRA, as PDGFRA expression was significantly downregulated after GSK3 inhibition. This is particularly interesting as PDGFRA overexpression in LGGs is induced by IDH mutation [39]. Our data suggests that PDGFRA expression in this context is dependent on GSK3 activity.

Notably, GSK3 inhibition enhanced proliferative capacity and cell cycling, as evidenced by an S phase increase. These results contrast previous observations made in glioblastoma [20], where GSK3 inhibition led to decreased proliferation, highlighting the biological distinction between IDH^mut^ and IDH^wt^ gliomas. This also shows that the effects of GSK3 activity are highly context-dependent as suggested in prior studies [49]. The accelerated proliferation of IDH^mut^ astrocytes under CHIR99021 treatment coincided with increased expression of RUNX2, identified through transcription factor enrichment analysis as a potential driver of the observed transcriptional changes. RUNX2 belongs to the highly conserved RUNX family of transcription factors [50]. It is a key player during bone development [43] and its activity is negatively regulated by GSK3β in osteoblasts [51]. Beyond osteogenesis, RUNX2 has been shown to foster tumor progression in osteosarcoma, gastric, pancreatic, and breast cancer, as well as promoting bone metastases [52–55]. As demonstrated in our study, knockdown of RUNX2 led to reduced clonogenicity and cell cycling with S phase decrease in GSK3-inhibited IDH^mut^ astrocytes. Furthermore, increased *RUNX2* expression in IDH^mut^ glioma patients correlated with significantly impaired survival, which has not been observed in glioblastoma patients. Notably, Yamada et al. [56] showed that expression of *RUNX2* increased from low- to high-grade gliomas, and correlated with enhanced proliferation, possibly through altered PKA signaling. While several findings by Yamada et al. match our observations, their study focused on IDH^wt^ tumors. Our results highlight the specific relationship between the IDH mutation in low-grade glioma, GSK3 expression, and RUNX2 target gene expression. These findings provide a basis for exploring this axis for low-grade gliomagenesis and as potential new therapeutic target in IDH^mut^ gliomas.

## Conclusions

Collectively, our study identifies GSK3 as a switch that determines the balance between oncogenic and migratory programs, and highlights its downstream effector RUNX2 as a transcription factor newly linked to poor prognosis in low-grade glioma. This study provides insights into the relevance of WNT/GSK3 signaling in early IDH^mut^ gliomagenesis and its presumptive downstream effector RUNX2, warranting further investigation into their translational applications.

## Electronic supplementary material

Below is the link to the electronic supplementary material.

Supplementary Material 1

Supplementary Material 2

Supplementary Material 3

Supplementary Material 4

Supplementary Material 5

## Data Availability

The datasets used and/or analyzed during the current study available from the corresponding author on reasonable request. The RNA-seq data supporting the conclusions of this article is deposited at GEO (GSE295254).

## Abbreviations

LGG: Low-grade glioma
NHA: Normal human astrocyte
IDH^mut^: Isocitrate Dehydrogenase mutation (IDH1^R132H^)
ECM: Extracellular matrix
FBS: Fetal bovine serum
DMSO: Dimethyl Sulfoxide
PFA: Paraformaldehyde
DAPI: 4‘,6-Diamidino-2-phenylindole
GO: Gene ontology
logFC: Log fold change
FDR: False discovery rate
CGGA: Chinese Glioma Genome Atlas
