# Supplementary figures and images for: GSK3 acts as a switch for transcriptional programs in a model of low-grade gliomagenesis
Source: Acta Neuropathol Commun. 2025 Apr 30;13:87. doi: 10.1186/s40478-025-02006-y (PMC12042597; doi:10.1186/s40478-025-02006-y)

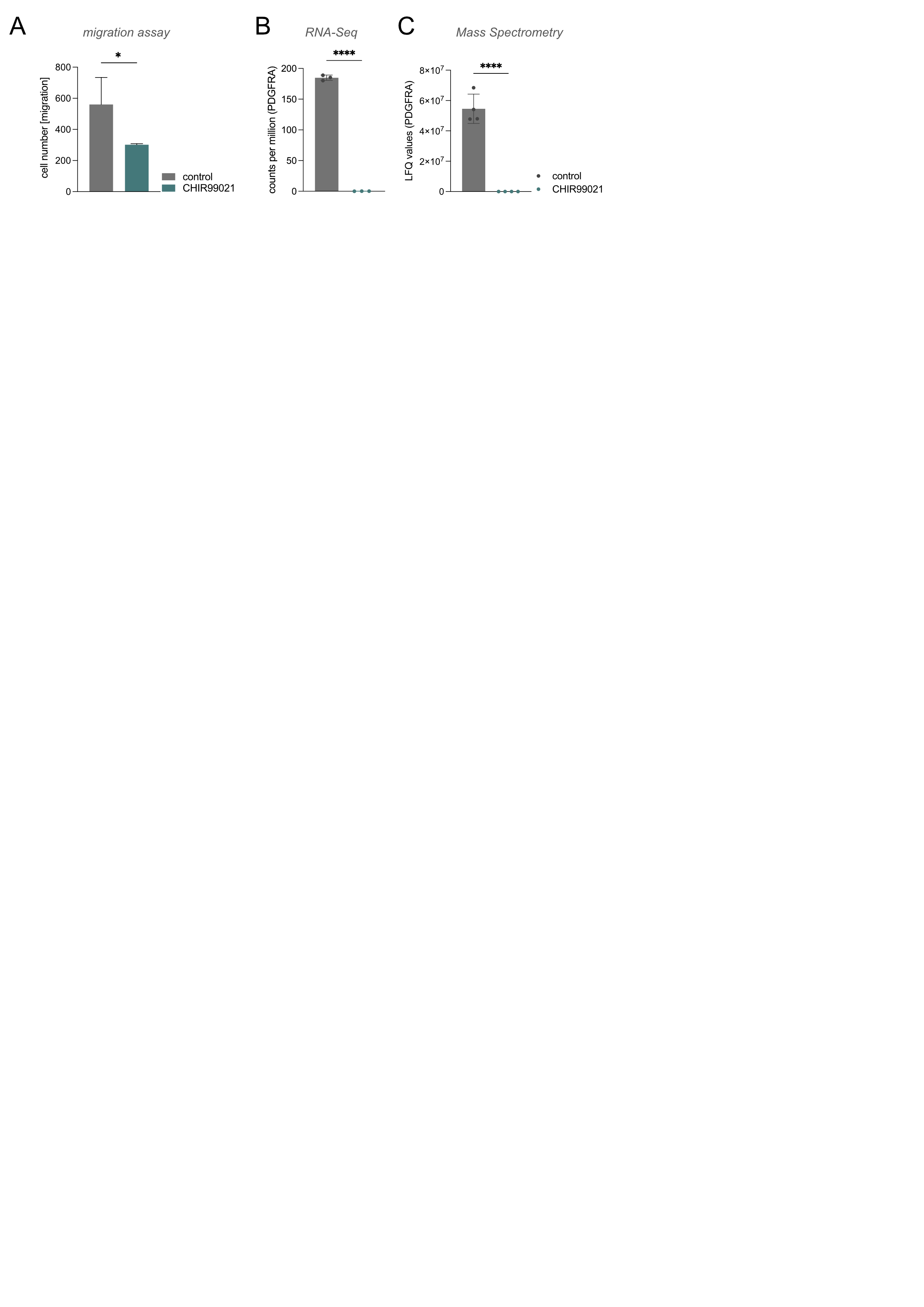

Supplement: Supplementary file 1 — Supplementary Material 1 [file 40478_2025_2006_MOESM1_ESM.tiff]

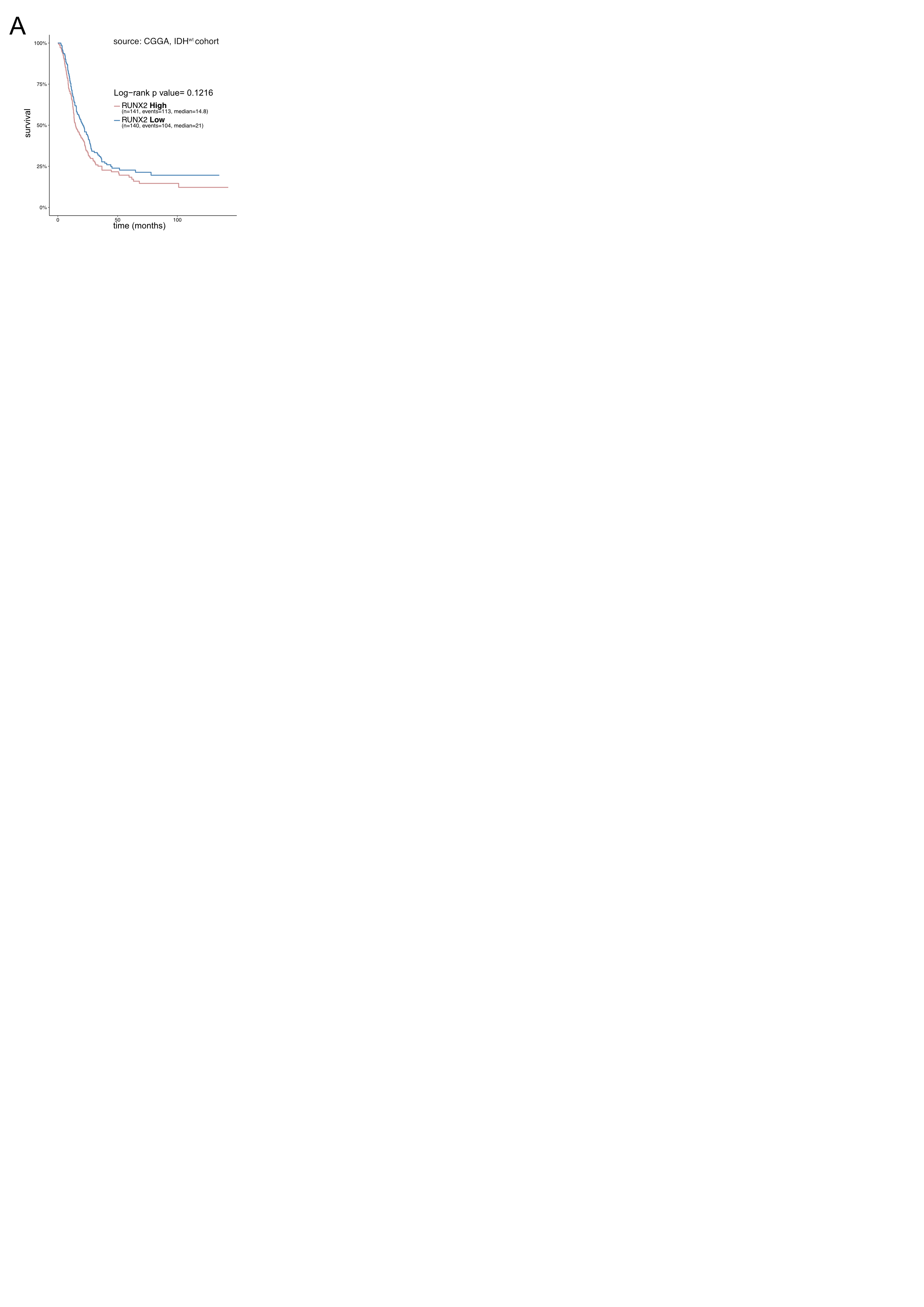

Supplement: Supplementary file 2 — Supplementary Material 2 [file 40478_2025_2006_MOESM2_ESM.tiff]

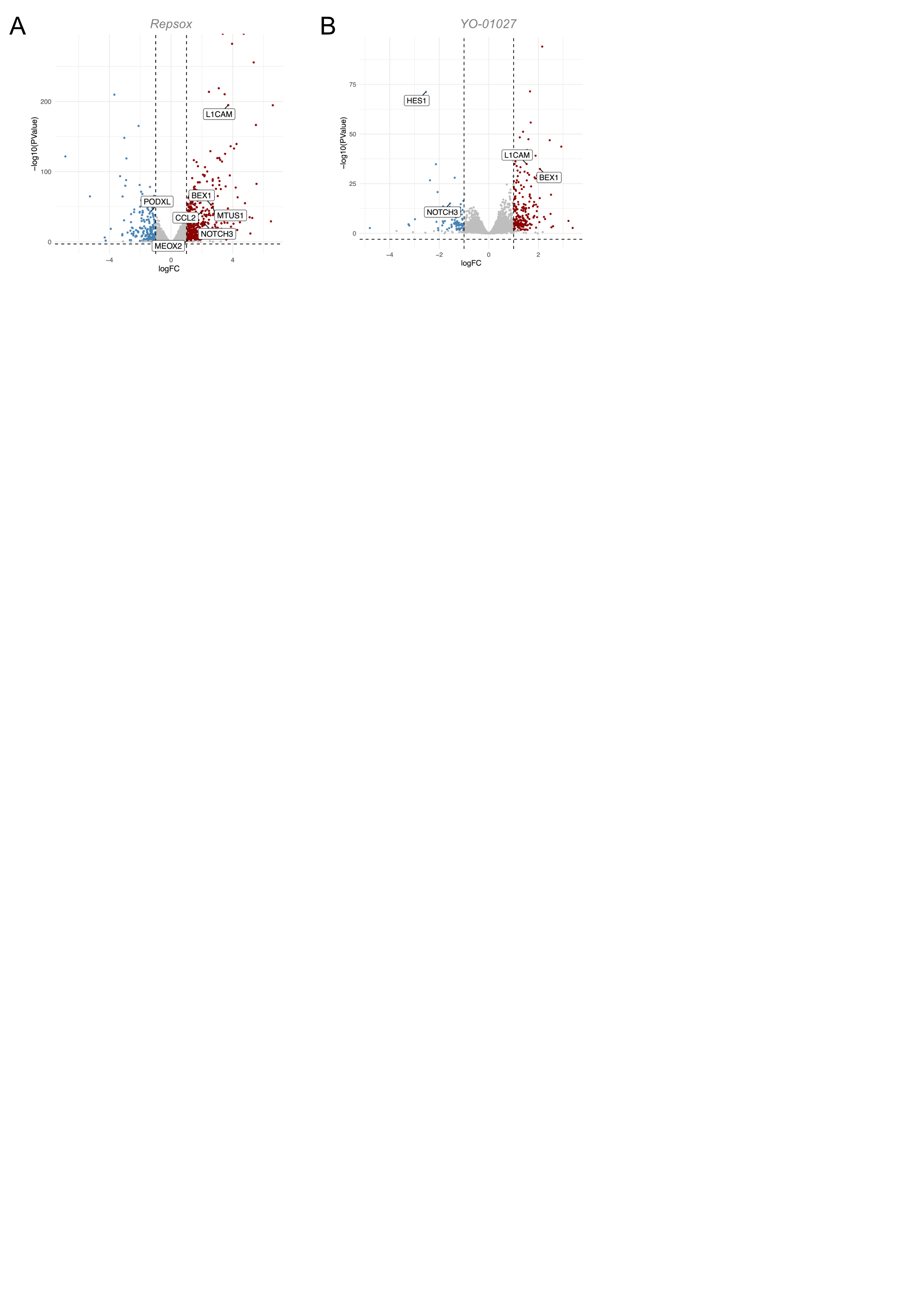

Supplement: Supplementary file 3 — Supplementary Material 3 [file 40478_2025_2006_MOESM3_ESM.tiff]
