# Supplementary material for: GSK3 acts as a switch for transcriptional programs in a model of low-grade gliomagenesis
Source: Acta Neuropathol Commun. 2025 Apr 30;13:87. doi: 10.1186/s40478-025-02006-y (PMC12042597; doi:10.1186/s40478-025-02006-y)
Supplement: Supplementary file 4 — Supplementary Material 4 [file 40478_2025_2006_MOESM4_ESM.docx]

**Supplementary Figure S1**

**A, B** Distribution of up- and downregulated DEGs after treatment with Repsox (A) and YO-01027 (B), with both treatments affecting transcription of only a limited fraction of genes compared to CHIR (YO-01027: up 193, down 78; Repsox: 471 up, 209 down), including LGG relevant gene sets.

**Supplementary Figure S2**

**A** Boyden-chamber based migration assay confirms reduced migration after GSK3 inhibition with CHIR99021 (p= 0.0101 – 0.0494, 1 biological replicate ns) (n= 3 biological and 5 technical replicates).

**B, C** CPM expression profiles (B) and LFQ expression profiles of PDGFRA from RNA-seq and mass spectrometry showing significant downregulation on both transcriptional and protein level.

**Supplementary Figure S3**

**A** RUNX2 expression does not alter survival among patients with IDH^wt^ gliomas (source Gliovis/CGGA).
